# Bioactive Peptides from Sodium Caseinate Hydrolysate with High Oral Absorption Regulate Blood Glucose in Type 2 Diabetic Mice via Inhibition of DPP-IV and Stimulation of GLP-1

**DOI:** 10.3390/foods14111953

**Published:** 2025-05-30

**Authors:** Pei-Yu Wu, Cheng-Hong Hsieh, Ali Iqbal, Yu-Shun Lin, Ming-Wei Cheng, Ling-Hsuan Chang, Shang-Ming Huang, Kuo-Chiang Hsu

**Affiliations:** 1 Department of Nutrition, China Medical University, 100 Sec. 1, Jingmao Road, Taichung 406040, Taiwan; peiyuwu@mail.cmu.edu.tw (P.-Y.W.); hyweuan@asia.edu.tw (C.-H.H.); alifoodtechnologist007@gmail.com (A.I.); yslin@mail.cmu.edu.tw (Y.-S.L.); zazamj2323@gmail.com (M.-W.C.); u111076006@cmu.edu.tw (L.-H.C.); 2 Department of Food Nutrition and Health Biotechnology, Asia University, 500 Lioufeng Road, Taichung 413305, Taiwan

**Keywords:** type 2 diabetes mellitus, sodium caseinate hydrolysate, dipeptidyl peptidase-IV, glucagon-like peptide-1

## Abstract

Type 2 diabetes mellitus remains a critical global health challenge, driving the pursuit of novel therapeutic strategies. This study investigated the anti-diabetic efficacy of the peptide 1CBR, derived from sodium caseinate hydrolysate, administered orally at 25 mg/kg/day to db/db mice over a 4-week period. Glucose tolerance was evaluated via oral glucose tolerance tests (OGTT), while plasma dipeptidyl peptidase-IV (DPP-IV) activity, glucagon-like peptide-1 (GLP-1), and insulin concentrations were quantified using enzyme-linked immunosorbent assays (ELISA). Two bioactive peptides, GPFPLPD and APDSGNFR, were isolated and characterized, exhibiting half-maximal inhibitory concentrations (IC_50_) of 99.12 µM and 73.07 µM for DPP-IV inhibition, respectively, and both significantly stimulated GLP-1 secretion in enteroendocrine cells in vitro. Pharmacokinetic analysis in Sprague–Dawley rats demonstrated oral bioavailability of 11.28% and 19.12% for these peptides, surpassing typical expectations for peptide-based agents. Collectively, these results provide compelling evidence that 1CBR-derived peptides exert glucose-lowering effects through the dual mechanisms of DPP-IV inhibition and GLP-1 stimulation, combined with favorable oral absorption profiles. These findings underscore the potential of 1CBR peptides as promising candidates for development into nutraceuticals or pharmaceutical agents for diabetes management.

## 1. Introduction

Type 2 diabetes (T2D) is a metabolic disease and one of the most rapidly increasing global health concerns [1]. Over the past decade, incretin-based therapy has emerged as a promising approach to diabetes management [2]. Incretins are gastrointestinal hormones that play a crucial role in maintaining glucose homeostasis. Among them, glucagon-like peptide-1 (GLP-1) stimulates insulin secretion in response to food intake, lowering blood glucose levels [3]. However, GLP-1 undergoes rapid degradation by dipeptidyl peptidase-IV (DPP-IV), which removes its N-terminal dipeptides, resulting in the loss of its insulin-stimulating function [4]. Therefore, DPP-IV inhibitors have been developed to prevent GLP-1 degradation and are widely used to treat T2D [5].

Several DPP-IV inhibitors, including alogliptin, linagliptin, saxagliptin, and sitagliptin, have been authorized for treating T2D in the European Union, the United States, and Japan. However, in 2015, the U.S. Food and Drug Administration (FDA) warned that these medications may cause severe and disabling joint pain based on the FDA Adverse Event Reporting System data and medical literature [6,7]. Consequently, there is an increasing need to develop naturally derived DPP-IV inhibitors with fewer adverse effects as potential therapeutic agents for T2D [5].

Previous studies worldwide have extensively explored the potential of natural bioactive peptides as DPP-IV inhibitors for diabetes management. For instance, peptides derived from various food proteins such as milk [8], fish [9], and soy [10] have demonstrated promising in vitro DPP-IV inhibitory activity and glucose-lowering effects in vivo. Moreover, certain peptides not only inhibit DPP-IV but also stimulate GLP-1 secretion, enhancing their therapeutic potential [11]. Notably, the oral bioavailability of these peptides remains a significant challenge due to enzymatic degradation and poor absorption, which limits their clinical translation [12]. Previous studies have demonstrated the anti-diabetic effects of protein hydrolysates derived from gelatin and sodium caseinate in diabetic rodent models [13,14]. Glycemic control in Streptozotocin (STZ)-induced diabetic rats has been improved through oral administration of gelatin hydrolysate derived from the skin of Atlantic salmon, the <1 kDa segment of gelatin hydrolysate from porcine skin, and the <1.5 kDa fractions from gelatin hydrolysates of tilapia and halibut skin over 35, 42, and 28 days, respectively [13,15,16]. In addition, the sodium caseinate hydrolysate (<1 kDa fraction) was also demonstrated to exhibit an anti-diabetic effect on rats induced by cotreatment with a high-fat diet (HFD) and low-dose STZ following six weeks of oral administration [14]. These studies suggest that peptides within these protein hydrolysates function as DPP-IV inhibitors, helping maintain intact GLP-1, promoting insulin secretion, and enhancing glycemic control.

Although the beneficial effects of protein hydrolysates in diabetic rats have been reported [13,14], three critical issues remain to be addressed. First, rodent models induced by chemical agents, such as high-dose STZ and multiple low-dose STZ, are typically used to simulate type 1 diabetes (T1D) by inducing hyperglycemia and insulitis, respectively [17]. However, a T2D animal model is favorable for evaluating the anti-diabetic effect of DPP-IV inhibitors. Second, plasma levels of active GLP-1 may be affected by DPP-IV inhibition and/or GLP-1 release from intestinal L-cells [18]. While numerous studies have identified peptides with DPP-IV inhibitory activity [4], some have also reported that specific peptides can stimulate GLP-1 secretion [19,20]. Therefore, it is essential to determine whether DPP-IV inhibitory peptides also function as GLP-1 secretion stimulators in a T2DM model. Third, poor peptide bioavailability remains a significant concern, as systemic circulation is required for peptides to exert in vivo bioactivity [21]. Moreover, data on the bioavailability of bioactive peptides remain limited, and understanding their in vivo stability, absorption, and accessibility is critical.

This study aimed to evaluate the anti-diabetic effects of 1CBR, the <1 kDa fraction of bromelain-hydrolyzed sodium caseinate, in db/db mice, a well-established type 2 diabetes model. Additionally, we sought to isolate and characterize bioactive peptides from 1CBR with dual functions as DPP-IV inhibitors and GLP-1 secretion stimulators in enteroendocrine cell lines. Finally, the oral bioavailability and systemic absorption of these peptides were assessed in Sprague–Dawley rats to determine their physiological relevance and potential for in vivo efficacy. Through this comprehensive approach, we aim to provide robust evidence supporting the therapeutic potential of 1CBR-derived peptides as natural alternatives for glycemic control and diabetes management.

## 2. Materials and Methods

### 2.1. Materials

Sodium caseinate was obtained from Gemfont Inc. (Taipei, Taiwan). Bromelain, derived from pineapple stem, was obtained from STBIO Media Inc. (New Taipei, Taiwan). Dipeptidyl peptidase-IV (DPP-IV; D7052, sourced from porcine kidney), Gly-Pro-p-nitroanilide, HEPES sodium salt (H3784), and RPMI-1640 medium (R8758) were obtained from Sigma-Aldrich (St. Louis, MO, USA). The remaining chemicals and reagents were of analytical grade and were available from commercial suppliers.

### 2.2. 1CBR (<1 kDa Fraction of the Hydrolysate of Sodium Caseinate by Bromelain) Preparation

1CBR was prepared following the procedure described in our previous study [14]. Briefly, sodium caseinate was mixed with a 25-fold volume (*w*/*v*) of ddH_2_O and hydrolyzed at pH 6.7 and 45 °C with bromelain (5% enzyme/substrate ratio) for 60 min. The resulting hydrolysate was then separated by ultrafiltration (Model ABL085, Lian Sheng Tech. Co., Taichung, Taiwan) with a spiral-wound membrane having a molecular mass cutoff of 1.0 kDa. The hydrolysate permeate was freeze-dried and kept in a desiccator until required.

### 2.3. Antidiabetic Effect of 1CBR in db/db Mice

#### 2.3.1. Animal Model and Diet

In this study, db/db mice (BKS.Cg-Dock7m+/+Leprdb/JNarl from National Laboratory Animal Center, Taipei, Taiwan) 5 weeks of age and weighing 22–25 g were analyzed. The mice were given the 5001-Laboratory Rodent Diet (LabDiet^®^, St. Louis, MO, USA), which comprised 57% carbohydrate, 13% fat, and 30% protein by total kcal. The Institutional Animal Care and Use Committee (IACUC) of China Medical University approved all animal care and experimental protocols.

#### 2.3.2. Diabetes Induction and Experimental Groups

Between the ages of 6 and 8 weeks, the blood glucose concentrations in the mice were monitored weekly. Diabetes was determined in mice when the blood glucose levels surpassed 400 mg/dL after an OGTT test (oral glucose tolerance). The mice were randomly allocated to three groups, each consisting of 13 individuals. The experimental period lasted for 3 weeks. Group C: control mice; Group P: mice administered 1CBR (500 mg/kg/day); Group S: mice administered sitagliptin (20.5 mg/kg/day; positive control). Mice were housed in groups of 3–4 per cage. The sample size was determined based on previous studies and ethical considerations (3Rs principle) to achieve adequate statistical power without excessive animal use. The dose of 1CBR (500 mg/kg/day) was selected based on our previous study [13], in which this concentration significantly improved glycemic control in HFD/STZ-induced diabetic rats without observed toxicity. This dose was also within the effective range reported for other food-derived bioactive protein hydrolysates in rodent models.

#### 2.3.3. OGTT Procedure

An OGTT was conducted on mice fasted overnight from each group at week 0 and week 3. Blood samples were collected by retro-orbital bleed at 0 to 120 min following a glucose challenge (2 g/kg) to determine glucose levels using a Glucose GOD-PAP kit (Randox Laboratories Ltd., Crumlin, UK).

#### 2.3.4. Blood Collection and Biochemical Assays

At week 3, blood samples were obtained from all mice into EDTA-treated chilled tubes and then centrifuged at 3000× *g* for 15 min. The supernatant was kept on ice for up to 3 h for further analysis. Plasma insulin levels and DPP-IV activity were determined using an insulin ELISA kit (Mercodia AB, Uppsala, Sweden) and a DPP-IV assay kit (Enzo Inc., Farmingdale, NY, USA), respectively. Plasma total GLP-1 levels were measured using the glucagon-like peptide-1 (total) kit from RayBiotech Inc. (Norcross, GA, USA), while active GLP-1 levels were assessed with the active GLP-1 kit from Millipore Corp. (Billerica, MA, USA), each from a different supplier.

### 2.4. Identification, Synthesis, and Evaluation of DPP-IV Inhibitory Peptides from 1CBR

#### 2.4.1. Purification of 1CBR Hydrolysates

In this study, 1CBR was purified by RP-HPLC using a Model L-2130 HPLC system (Hitachi Ltd., Katsuda, Japan). Afterward, 2 mg of the lyophilized hydrolysate fraction was reconstituted in 1 mL of ddH_2_O, and 60 μL of the resulting solution was subjected to injection onto a ZORBAX Eclipse Plus C18 column (4.6 mm × 250 mm, Agilent Tech. Inc., Santa Clara, CA, USA) for analysis. A gradient of acetonitrile, ranging from 5% to 60% over 60 min, was applied in 0.1% TFA at 0.7 mL/min. Peptides were monitored at 220 nm. After collection, each fraction was lyophilized and stored in a desiccator for future use. The fractions were then re-dissolved in 100 mM Tris buffer (pH 7.0) to achieve a 1 mg/mL concentration and were evaluated for in vitro DPP-IV inhibitory activity. The assay for DPP-IV inhibition was conducted following the procedure outlined in our previous study [18].

#### 2.4.2. Peptide Identification via MALDI-TOF/TOF MS

The fraction exhibiting the strongest in vitro DPP-IV inhibitory activity was subjected to peptide analysis using a MALDI-TOF/TOF mass spectrometer (UltraFlex III, Bruker Daltonics, Billerica, MA, USA) equipped with a delayed extraction source and a 335 nm pulsed nitrogen laser. A 0.6 μL aliquot of peptide solution (1 mg/mL) was mixed with an equal volume of saturated α-cyano-4-hydroxycinnamic acid matrix and then air-dried on the target plate. The dried sample was subsequently analyzed using MALDI-TOF/TOF MS in positive ion reflection mode under a 20 kV accelerating voltage. Mass spectra were generated by averaging 200 laser shots within an m/z range of 200–2500 Da. Tandem mass spectrometry (MS/MS) was performed to determine peptide sequences, with data processed using BioTools software (Version 3.2, Bruker Daltonics, Billerica, MA, USA).

#### 2.4.3. Peptide Synthesis and Activity Evaluation

The selected peptides were generated through Fmoc-based solid-phase peptide synthesis using an automated synthesizer (CS 136, CS Bio Co., San Carlos, CA, USA). Their structural integrity and purity were confirmed via RP-HPLC-MS/MS analysis. To assess their inhibitory potential against DPP-IV, the IC_50_ values were determined through in vitro enzymatic assays.

### 2.5. In Vitro GLP-1 Secretion Induced by DPP-IV Inhibitory Peptides

NCI-H716 and STC-1 enteroendocrine cell lines are widely conducted to investigate the mechanisms of GLP-1 expression. The STC-1 and NCI-H716 cell lines in this study were sourced from Bioresource Collection and Research (BCRC, Hsinchu, Taiwan). STC-1 cells were seeded in 12-well plates at 2 × 10^6^ cells/mL, while NCI-H716 cells were seeded in Matrigel-coated 12-well plates at 2 × 10^5^ cells/mL. Upon reaching 85% confluence, the supernatants were replaced with HEPES buffer for STC-1 and RPMI-1640 buffer without FBS for NCI-H716. Cells were treated with synthetic peptides at 2, 2.5, and 3 mM concentrations or sitagliptin at 2, 2.5, and 3 µM. After incubating at 37 °C with 5% CO_2_ for 3 h, the supernatants were harvested and assessed for GLP-1 levels using the active glucagon-like peptide-1 kit from Millipore Corp. (Billerica, MA, USA).

### 2.6. In Vivo Absorption of DPP-IV Inhibitory Peptides

#### 2.6.1. Animal Model and Experimental Design

In this study, GPFPLPD and APDSGNFR peptides were synthesized and prepared for the in vivo absorption study as outlined in Section 2.4. The experiment involved ten male Sprague–Dawley rats (LASCO, Taipei, Taiwan), each 6 weeks old and weighing between 230 and 250 g. Prior to the experiment, the rats were allowed a 7-day acclimatization period, during which they were fed a 5012-Rat Diet (LabDiet^®^, St. Louis, MO, USA), consisting of 60% carbohydrates, 13% fat, and 27% protein based on total caloric content. The rats were randomly assigned to two groups (5 rats per group) and orally administered a single dose of either GPFPLPD or APDSGNFR peptide at 16 mg/kg body weight.

#### 2.6.2. Blood Collection and Sample Preparation

Overnight-fasted rats were administered the peptides, and blood samples were collected from the tail vein at 0, 30, 60, 90, and 180 min post-administration. To obtain plasma, the samples were centrifuged at 3000× *g* for 15 min at 4 °C. A 500 µL aliquot of plasma was then combined with an equal volume of acetonitrile, followed by additional centrifugation at 1200× *g* for 10 min at 4 °C to precipitate high-molecular-weight proteins. The resulting supernatant (20 µL) was analyzed via RP-HPLC under the conditions outlined in Section 2.4. Peptide concentrations were quantified using calibration curves from peptide standards prepared in 50% (*v*/*v*) acetonitrile (Figure S4). The estimated blood volume of male rats was 6.86 mL per 100 g of body weight [22].

#### 2.6.3. Pharmacokinetic Analysis

The pharmacokinetic parameters, including the half-life (t_1/2_), area under the plasma drug concentration–time curve (AUC), time-to-peak concentration (T_max_), and peak concentration (C_max_), were directly determined and analyzed. The absorption ratio (AR_t_; %) was defined as the percentage of the administered peptide dose (16 mg/kg BW) present in plasma, calculated as the product of peptide concentration and blood volume at each time point. The AUC method was also employed to quantify peptide absorption. The fractional absorption (%) of orally administered peptides was calculated using the following formula [23]:

$$\mathrm{Fractional}\;\mathrm{absorption}\;(\%)=\frac{\ln2}{t_{1/2}}\times \frac{calculated\;oral\;AUC\times plasma\;volume}{oral\;dose}\times 100$$

To verify that the peaks observed in the RP-HPLC chromatogram corresponded to the intact peptides GPFPLPD and APDSGNFR, plasma samples from rats with or without peptide administration were analyzed by MALDI-TOF/TOF, as described in Section 2.4.2.

### 2.7. Statistical Analysis

Experimental results were expressed as the mean ± standard deviation (SD). Prior to parametric testing, the Shapiro–Wilk test was used to assess the normality of data distribution, as it is well-suited for small sample sizes (n = 13 per group). Statistical comparisons among groups were conducted using one-way analysis of variance (ANOVA), followed by Duncan’s multiple range test for post hoc comparisons. A *p*-value of less than 0.05 was considered statistically significant. All analyses were performed using SPSS software (version 12, IBM Corp., Armonk, NY, USA).

## 3. Results and Discussion

### 3.1. Antidiabetic Effect of 1CBR in db/db Mice

**Figure 1:**
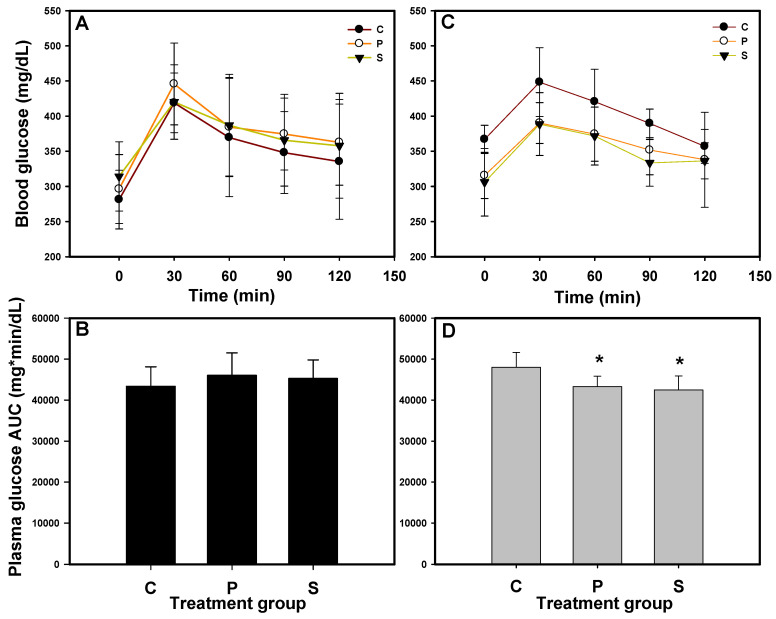
Oral glucose tolerance test (OGTT) results throughout the experimental period. (**A**) Blood glucose levels and (**B**) the area under the plasma drug concentration–time curve (AUC) at week 0. (**C**) Blood glucose levels and (**D**) AUC at week 3. Mice were administered glucose (2 g/kg), and blood glucose levels were monitored over 120 min. Group C: control mice; Group P: mice administered 1CBR (500 mg/kg/day); Group S: mice administered sitagliptin (20.5 mg/kg/day; positive control). Data are presented as the mean ± standard deviation. * Indicates *p* < 0.05 compared to the control group.

The OGTT was performed at weeks 0 and 3, and the blood glucose concentrations were monitored over 120 min. The area under the curve (AUC) was calculated to assess glucose tolerance, as shown in Figure 1. At week 0, the blood glucose concentrations in all the mice exceeded 400 mg/dL (Figure 1A), and mice with no significant differences in AUC (Figure 1B) were randomly assigned to 3 groups. After 3 weeks of administration (Figure 1C,D), mice in groups P (1CBR, 500 mg/kg/day) and S (sitagliptin, 20.5 mg/kg/day; positive control) exhibited significantly lower AUC values compared to those in group C (*p* < 0.05). Additionally, the mean plasma glucose concentrations in groups P and S were below 400 mg/dL, which we defined as the threshold for diabetes in mice. The findings align with our previous research [14], demonstrating that the administration of 1CBR at 500 mg/kg/d effectively reduces blood glucose levels in diabetic animal models, including db/db mice and HFD/STZ-induced diabetic rats.

**Figure 2:**
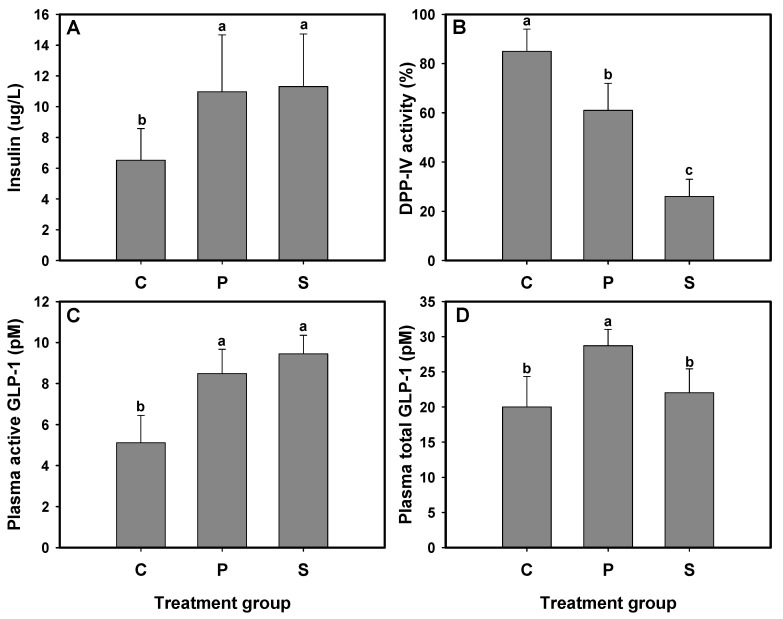
Effects of 1CBR peptide on glucose metabolism in db/db mice after 3 weeks of administration. (**A**) Plasma insulin levels. (**B**) Plasma DPP-IV activity. (**C**) Plasma active GLP-1 levels. (**D**) Plasma total GLP-1 levels. Data are presented as the mean ± standard deviation. Different letters indicate statistically significant differences (*p* < 0.05).

The effect of protein hydrolysates on glucose tolerance improvement has been attributed to increased insulin secretion, DPP-IV inhibition, and increased levels of active or total GLP-1 [13,15,16]. After the 3-week treatment period, plasma insulin levels, active and total GLP-1 concentrations, and DPP-IV activity were measured in db/db mice administered 1CBR (group P) or sitagliptin (group S). The results indicated that plasma insulin levels in db/db mice from groups P and S were significantly elevated, reaching 10.97 and 11.31 μg/L, respectively, compared to the untreated group C (6.51 μg/L) (Figure 2A). Additionally, plasma DPP-IV activity in groups P and S was reduced by 64% and 26%, respectively, which was notably lower than that in group C (83%) (*p* < 0.05) (Figure 2B). The active GLP-1 levels were also significantly increased, with concentrations of 5.22, 9.72, and 12.29 pM in groups C, P, and S, respectively, highlighting the effectiveness of both 1CBR and sitagliptin in elevating active GLP-1 levels (Figure 2C). Furthermore, the total GLP-1 level in group P was significantly higher (*p* < 0.05) than that in both groups C and S (Figure 2D).

Previous studies using C57BL/KsJ-db/db mice, which were administered silk fibroin and silk protein hydrolysates for 6 and 4 weeks, respectively, demonstrated that these two protein hydrolysates presented anti-hyperglycemic effects mainly by increasing pancreatic β-cell mass and enhancing insulin-releasing activity [24,25]. Our study has previously revealed that hydrolysates from fish and porcine skin gelatin act as DPP-IV inhibitors, exhibiting an anti-hyperglycemic effect in STZ-induced diabetic rats by inhibiting DPP-IV activity, increasing active GLP-1 levels, and stimulating insulin secretion [13,15,16]. DPP-IV inhibitors protect endogenous GLP-1 from degradation, allowing the remaining active GLP-1 to stimulate insulin secretion [26]. A previous study has reported that a zein hydrolysate increased total GLP-1 level in rats, accompanied by elevated active GLP-1 concentration and reduced plasma DPP-IV activity in the ileal vein [27]. Our previous study also indicated that 1CBR acts as a DPP-IV inhibitor, enhancing the secretion of active GLP-1 and improving glycemic control in HFD/STZ-induced diabetic rats [14]. While the animal models in both studies differed, the observed effects and underlying mechanisms of 1CBR in managing glycemic control remain consistent, reinforcing its potential for broader therapeutic application in diabetes treatment.

In summary, 1CBR demonstrates dual activity as a DPP-IV inhibitor and a GLP-1 secretion stimulator, thereby reducing blood glucose levels in db/db mice. This interpretation is supported by the data in Figure 2. DPP-IV activity was significantly decreased in group P (Figure 2B), while total GLP-1 levels were significantly elevated compared to both control and sitagliptin groups (Figure 2D), indicating enhanced secretion. Active GLP-1 levels (Figure 2C) reflect the combined effect of both mechanisms. These findings support the conclusion that 1CBR exerts its glucose-lowering effect through a dual mechanism involving both DPP-IV inhibition and stimulation of GLP-1 secretion.

### 3.2. Identification of the DPP-IV Inhibitory Peptides in 1CBR

To elucidate the bioactive peptides responsible for DPP-IV inhibition in 1CBR and their role in GLP-1 secretion (Figure 2D), 1CBR was subjected to HPLC fractionation, peptide sequencing, and subsequent synthesis. Initially, HPLC purification of the hydrolysate yielded five distinct fractions, each assessed for DPP-IV inhibitory activity (Figure S1). The inhibition rates ranged from 31.8% (F5) to 56.2% (F2) at 0.5 mg/mL, F2 demonstrating the highest inhibitory potential. The most active fraction (F2) yielded four major peptides identified via MALDI-TOF MS: GPFPLPD, GGKPSSMT, GGHLFFC, and APDSGNFR (Figure S2). These peptides were synthesized and further evaluated for their DPP-IV inhibition and ability to stimulate GLP-1 secretion. Enzymatic inhibition kinetics are depicted in Figure S3, and IC_50_ values are summarized in Table 1. Notably, GPFPLPD and APDSGNFR, characterized by a proline residue at the second N-terminal position, exhibited significantly lower IC_50_ values (99.12 and 73.07 µM, respectively) compared to GGKPSSMT and GGHLFFC, which contained glycine at the same position and displayed IC_50_ values exceeding 600 µM (Table 1). These findings corroborate previous observations that peptides harboring alanine or proline at the penultimate N-terminal position exhibit potent DPP-IV inhibitory effects [24]. Consequently, GPFPLPD and APDSGNFR were identified as the most promising DPP-IV inhibitors and were selected for further investigation [28].

### 3.3. Effect of 1CBR Peptides on GLP-1 Secretion in NCI-H716 and STC-1

To investigate the impact of 1CBR-derived peptides on GLP-1 secretion, the synthesized peptides APDSGNFR and GPFPLPD were evaluated using NCI-H716 and STC-1 cell models. The NCI-H716 cell line serves as a well-established human model for studying GLP-1 secretion mechanisms [29], while STC-1 cells, exhibiting enteroendocrine L-cell-like properties, are commonly employed for the in vitro screening of food-derived modulators of gastrointestinal hormone secretion [30].

**Figure 3:**
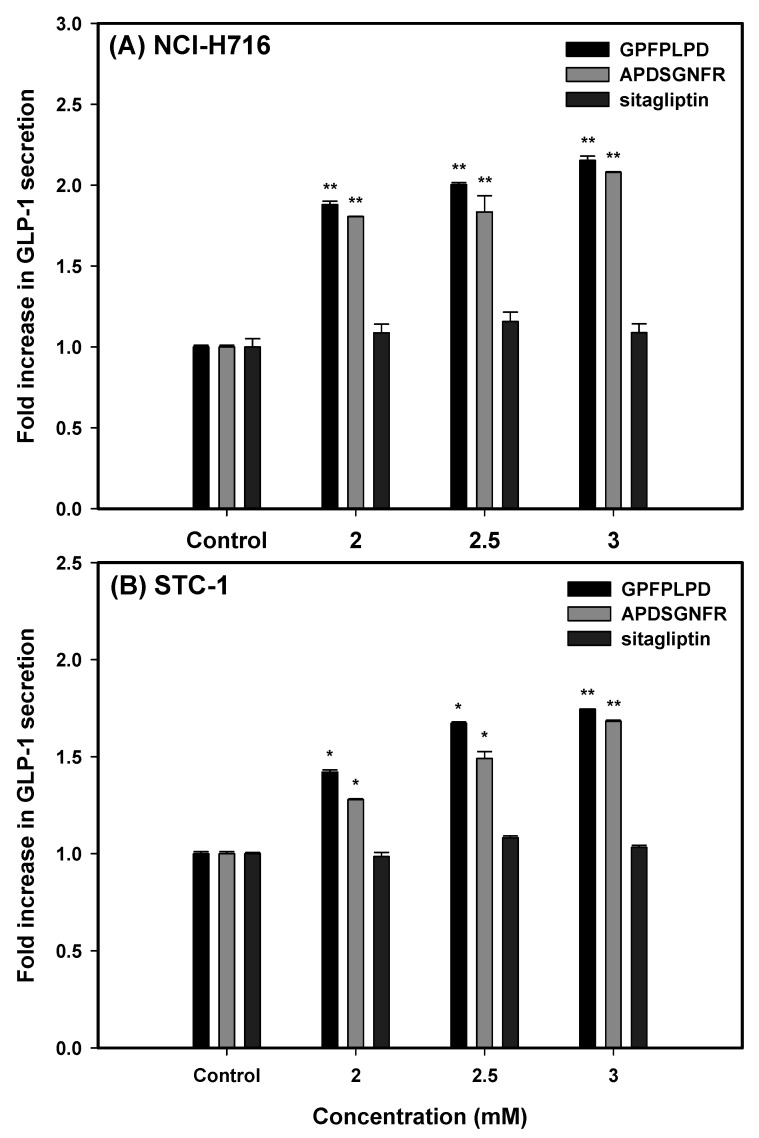
Effect of synthetic peptides APDSGNFR and GPFPLPD derived from 1CBR on GLP-1 secretion in (**A**) NCI-H716 and (**B**) STC-1 cells. Both cells were incubated with APDSGNFR, GPFPLPD, or sitagliptin at concentrations of 2.0, 2.5, and 3.0 mM for 3 h. GLP-1 secretion in the culture supernatant was quantified. Bars represent the means ± standard deviations from three independent experiments. * Indicates *p* < 0.05, ** Indicates *p* < 0.01 compared to the control group.

Our findings revealed a significant, dose-dependent enhancement in GLP-1 secretion (*p* < 0.01) following a 3 h incubation of both cell lines with increasing concentrations (2.0, 2.5, and 3.0 mM) of the two peptides or sitagliptin (Figure 3). In NCI-H716 cells, treatment with GPFPLPD led to a 1.88- to 2.15-fold increase in GLP-1 secretion relative to untreated controls, while APDSGNFR elicited a 1.81- to 2.08-fold increase. A similar trend was observed in STC-1 cells, where GPFPLPD administration at 2.0, 2.5, and 3.0 mM resulted in a respective 42.1%, 67.2% (*p* < 0.05), and 74.6% (*p* < 0.01) rise in GLP-1 secretion. APDSGNFR exhibited a comparable but slightly attenuated effect. Notably, sitagliptin treatment did not influence GLP-1 secretion in either cell model.

Prior studies have demonstrated that meat hydrolysates (MH) stimulate GLP-1 secretion in rodent-derived L-cells [31] and NCI-H716 cells [29]. These effects have been linked to the activation of the ERK1/2 MAPK signaling pathway, with further enhancement observed upon supplementation with essential amino acids, which also activates p38 MAPK [32]. Additionally, peptide structural composition is critical in modulating GLP-1 release in STC-1 cells, as these cells selectively recognize specific protein fragments [33,34]. However, compared to the bioactive peptides, sitagliptin failed to induce GLP-1 secretion in either cell line, potentially explaining the unchanged plasma total GLP-1 concentrations in db/db mice observed in the S group (Figure 2D).

In our current study, the observed elevation of GLP-1 levels following treatment with 1CBR-derived peptides may be attributed to both reduced enzymatic degradation and potentially enhanced synthesis of GLP-1. While the inhibitory effect on DPP-IV activity provides a plausible explanation for the increased stability and prolonged half-life of active GLP-1 in plasma and cell culture models, we cannot exclude the possibility that these peptides also stimulate de novo synthesis or secretion of GLP-1 from enteroendocrine cells. Future investigations incorporating gene expression analysis of key components in the GLP-1 biosynthetic pathway (e.g., *proglucagon* gene expression in L-cells) will be essential to clarify whether the upregulation of GLP-1 synthesis contributes to the observed effects.

### 3.4. Absorption of DPP-IV Inhibitory Peptides

**Figure 4:**
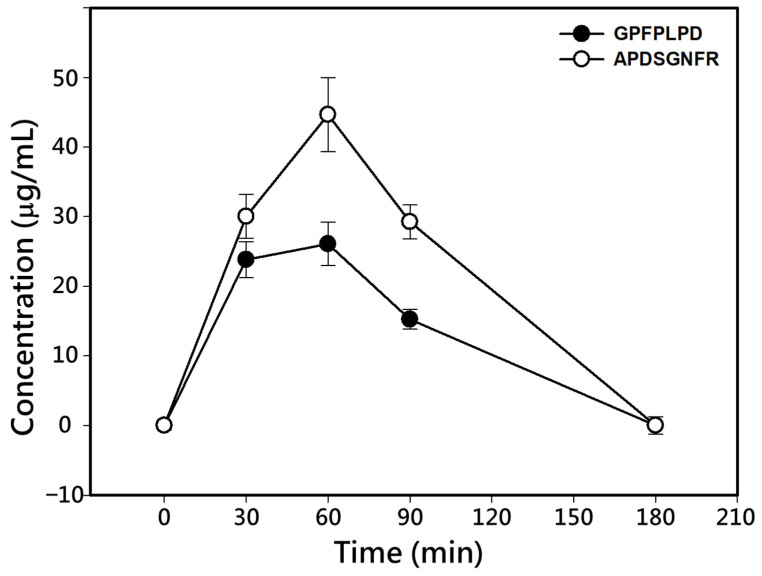
Plasma concentration profiles of DPP-IV inhibitory peptides following oral administration in rats. Sprague–Dawley rats were orally administered a single dose of 16 mg/kg BW of APDSGNFR or GPFPLPD, and peptide concentrations in plasma were determined at 0, 30, 60, 90, and 180 min using RP-HPLC. The selected dose was intended to ensure detectable plasma concentrations while minimizing peptide degradation or receptor saturation, representing a practical compromise between physiological relevance and analytical sensitivity. Data are presented as the mean ± standard deviation.

To evaluate the absorption of DPP-IV inhibitory peptides, Sprague–Dawley rats were orally administrated a single dose of 16 mg/kg BW of each synthesized peptide, APDSGNFR or GPFPLPD. The peptide concentrations in blood at 0, 30, 60, 90, and 180 min post-administration were determined using RP-HPLC analysis (Figure 4). The peptides in plasma were identified and quantified by comparing their retention times (Figure S4) and the calibration curves established previously (Figure S5).

**Figure 5:**
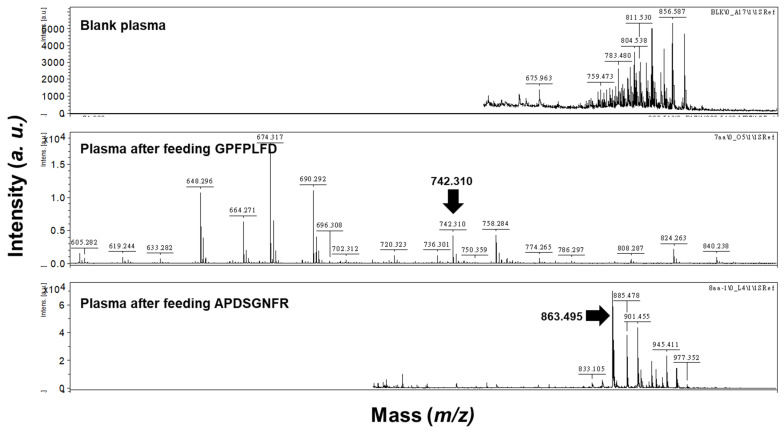
MALDI-TOF/TOF mass spectra of APDSGNFR and GPFPLPD detected in rat plasma at 60 min post-administration. Peptides were analyzed in positive ion reflection mode with a source voltage of 20 kV. Spectra were acquired by averaging 200 shots within the *m/z* range of 200–2500 Da.

The kinetic parameters of the two peptides are summarized in Table 2. Both peptides exhibited a marked increase in plasma concentrations within the first 60 min post-administration, followed by a decline, returning to baseline levels by 180 min (Figure 4). The maximum concentration (C_max_) for GPFPLPD and APDSGNFR was observed at 60 min, with values of 26.08 and 44.63 µg/mL, respectively. The area under the concentration–time curve (AUC) for the two peptides was 2411 ± 396 and 3994 ± 668 μg/mL plasma, respectively, and their elimination half-life time (t_1/2_) was 98.7 min for GPFPLPD and 109.2 min for APDSGNFR (Table 2). The absorption ratio of GPFPLPD and APDSGNFR increased within the first 60 min, reaching peak levels of 11.28 and 19.12%, respectively. Afterward, the absorption ratios gradually decreased, returning to baseline at 180 min. The fractional absorption, calculated based on the AUC, was 7.2% for GPFPLPD and 10.8% for APDSGNF. MALDI-TOF/TOF analysis confirmed that both APDSGNFR and GPFPLPD remained structurally intact in plasma at 60 min post-administration (Figure 5). The data indicated that no peptides were detected in the plasma. At the same time, GPFPLPD and APDSGNFR were identified in plasma after administration, confirming that these peptides resisted gastrointestinal digestion and entered the bloodstream.

Prior research has shown that only a tiny fraction of orally administered peptides successfully traverses the intestinal epithelium. Peptides that enter circulation bind to plasma proteins and are susceptible to further degradation by blood peptidases before exerting their physiological effects [35,36]. For instance, an antihypertensive casein-derived peptide, HLPLP, was administered at 40 mg/kg BW in adult Wistar rats, and it showed an absolute bioavailability of 5.18% with a low absorption rate (C_max_ of 20.1 ng/mL reached at 11.8 min) [37]. A separate investigation examined the pharmacokinetic profiles of proline-rich tripeptides in pigs following intravenous injection or intragastric delivery at a dosage of 4.0 mg/kg body weight in saline. The fraction of dose absorbed was approximately 0.059–0.077%, with an absolute bioavailability of about 0.1% and C_max_ values of 9–12 nmol/L following intragastric dosing [38]. Likewise, in a rat model, the antihypertensive peptide IRW exhibited a bioavailability of 11.7%, with elimination half-lives of 7.9 min following oral administration and 28.5 min after intravenous injection [39]. The antioxidative peptide WDHHAPQLR exhibited a bioavailability of 3.56% in rats after a single oral dose of 100 mg/kg body weight, with an elimination half-life of 1.19 h [40].

While the anti-diabetic effects of 1CBR and its constituent peptides are clearly demonstrated in rodent models, the translation of these findings to human physiology requires careful consideration. Variations in gastrointestinal digestion, peptide transport, and DPP-IV expression between species may significantly impact the bioavailability and efficacy of dietary peptides in humans. In particular, although GPFPLPD and APDSGNFR exhibited potent in vitro bioactivities, including DPP-IV inhibition and GLP-1 stimulation, the relevance of these sequences in vivo must be interpreted with caution. Peptides derived from dietary proteins are often further hydrolyzed during digestion, and their intact presence in systemic circulation is not guaranteed. However, in our absorption study, both GPFPLPD and APDSGNFR were detected in plasma 60 min post-oral administration, indicating partial resistance to proteolysis and supporting their potential physiological role.

Nonetheless, it remains possible that other digestion-derived peptides not evaluated in this study also contribute to the observed in vivo effects. Therefore, future research should incorporate comprehensive peptidomic profiling of gastrointestinal and circulating peptides following 1CBR administration to fully elucidate the bioactive components responsible for the antidiabetic effects. In addition, given the short half-lives of these peptides, formulation strategies to enhance their stability or enable controlled release may be necessary for effective application. Human-relevant models such as intestinal organoids, pilot clinical trials, and dose optimization studies will be essential next steps to evaluate safety, efficacy, and pharmacokinetics in a translational context, ultimately informing the feasibility of developing 1CBR-derived peptides into functional foods or nutraceutical interventions for type 2 diabetes.

## 4. Conclusions

DPP-IV is an enzyme that degrades GLP-1, a crucial incretin hormone involved in insulin secretion and glucose regulation, positioning DPP-IV inhibition as a key therapeutic target for diabetes. In this study, oral administration of 1CBR (a sub-1 kDa fraction derived from sodium caseinate hydrolyzed by bromelain) at 500 mg/kg/day to db/db mice improved glycemic control. These improvements included reductions in blood glucose levels, enhanced insulin secretion, reduced plasma DPP-IV activity, and increased concentrations of active GLP-1. These findings align with our earlier research in HFD/STZ-induced type 1 diabetic rats, supporting the anti-diabetic potential of 1CBR. HPLC fractionation and MALDI-TOF MS analysis identified GPFPLPD and APDSGNFR as the most potent DPP-IV inhibitors, with IC_50_ values of 99.12 µM and 73.07 µM, respectively, and favorable oral bioavailability, showing absorption ratios of 11.28% and 19.12%. Additionally, in vitro experiments with enteroendocrine cell models, NCI-H716 and STC-1, confirmed that both peptides effectively promoted GLP-1 secretion. Taken together, these findings suggest that the anti-diabetic effects of 1CBR are mediated through direct DPP-IV inhibition and stimulation of GLP-1 secretion, primarily attributed to the bioactive peptides GPFPLPD and APDSGNFR, making 1CBR a promising natural therapeutic candidate for type 2 diabetes. Future studies should prioritize clinical validation of its therapeutic efficacy, elucidate the underlying molecular mechanisms, and assess its potential integration into functional foods and diabetes management strategies.

Despite the promising results, several limitations should be acknowledged. First, although db/db mice are a well-established model of type 2 diabetes, their metabolic physiology and peptide absorption profiles differ from those of humans, which may limit the direct translatability of the findings. Second, the bioavailability of orally administered peptides can be influenced by factors such as gastrointestinal degradation and interspecies variability in peptide transport mechanisms. Third, while in vitro cell models confirmed GLP-1 secretion, further elucidation of receptor pathways and signaling cascades is needed to clarify the underlying molecular mechanisms.

## Figures and Tables

**Table 1:** Peptide sequences identified by mass spectrometry and their inhibitory activity against DPP-IV.

Peptide Sequence	*m*/*z*	Mean Mass	IC50 (µM)
GPFPLPD	742.327	741.320	99.12
GGKPSSMT	764.292	763.285	838.66
GGHLFFC	780.276	779.269	642.07
APDSGNFR	863.326	862.319	73.07

**Table 2:** Pharmacokinetic parameters for bioactive peptides GPFPLPD and APDSGNFR following a single oral dose of 16 mg/kg BW in rats.

	Bioactive Peptides	
Parameters	GPFPLPD	APDSGNFR
C_max_ (μg/mL plasma)	26.08 ± 4.12	44.63 ± 9.33
T_max_ (min)	60	60
t_1/2_ (min)	98.7	109.2
AUC (μg·min/mL plasma)	2411 ± 396	3994 ± 668
AR_30_ (%)	10.21 ± 1.48	12.74 ± 2.17
AR_60_ (%)	11.28 ± 1.89	19.12 ± 3.51
AR_90_ (%)	6.52 ± 0.66	12.40 ± 1.97
Fractional absorption (%)	7.2	10.8

Data are the mean ± SD (n = 5). C_max_, maximum plasma concentration; T_max_, peak concentration; t_1/2_, half-life; AUC, area under the curve; AR_t_, absorption ratio at the determined time.

## Data Availability

The original contributions presented in the study are included in the article/Supplementary Material, further inquiries can be directed to the corresponding author.

## Supplementary Materials

The following supporting information can be downloaded at: https://www.mdpi.com/article/10.3390/foods14111953/s1, Figure S1: HPLC purification of the protein hydrolysate yielded five distinct fractions (F1–F5). Different letters (a, b, c, d) above the bars indicate statistically significant differences between groups; Figure S2: MALDI-TOF MS analysis of Fraction 2 (F2) revealed four predominant peptide sequences: GPFPLPD, GGKPSSMT, GGHLFFC, and APDSGNFR; Figure S3: Enzymatic inhibition kinetics of DPP-IV in the presence of synthesized peptides; Figure S4: The elution profiles of the synthetic peptides GPFPLPD (designated as peak a) and APDSGNFR (designated as peak b) were characterized by reversed-phase high-performance liquid chromatography (RP-HPLC); Figure S5: Calibration curves of the synthetic peptides GPFPLPD and APDSGNFR for quantitative analysis in the rat absorption study.
