# Supplementary material for: Bioactive Peptides from Sodium Caseinate Hydrolysate with High Oral Absorption Regulate Blood Glucose in Type 2 Diabetic Mice via Inhibition of DPP-IV and Stimulation of GLP-1
Source: Foods. 2025 May 30;14(11):1953. doi: 10.3390/foods14111953 (PMC12154214; doi:10.3390/foods14111953)
Supplement: Supplementary file 1 [file foods-14-01953-s001.zip › foods-3634883-supplementary.pdf]

Supplementary Materials

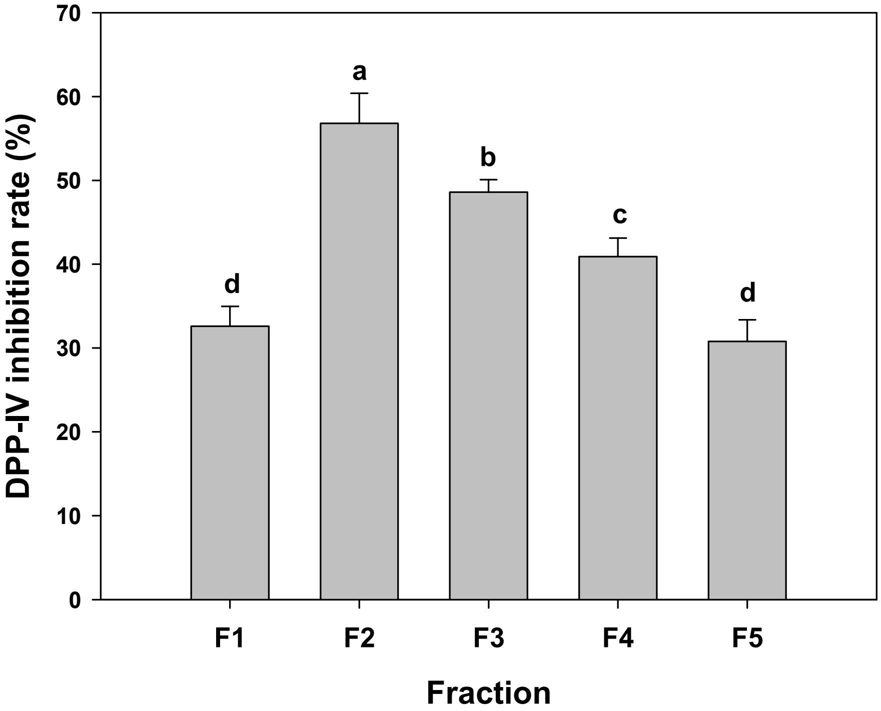

Figure. S1. HPLC purification of the protein hydrolysate yielded five distinct fractions (F1–F5). Each fraction was collected based on UV absorbance at 220 nm and subsequently assessed for DPP-IV inhibitory activity.

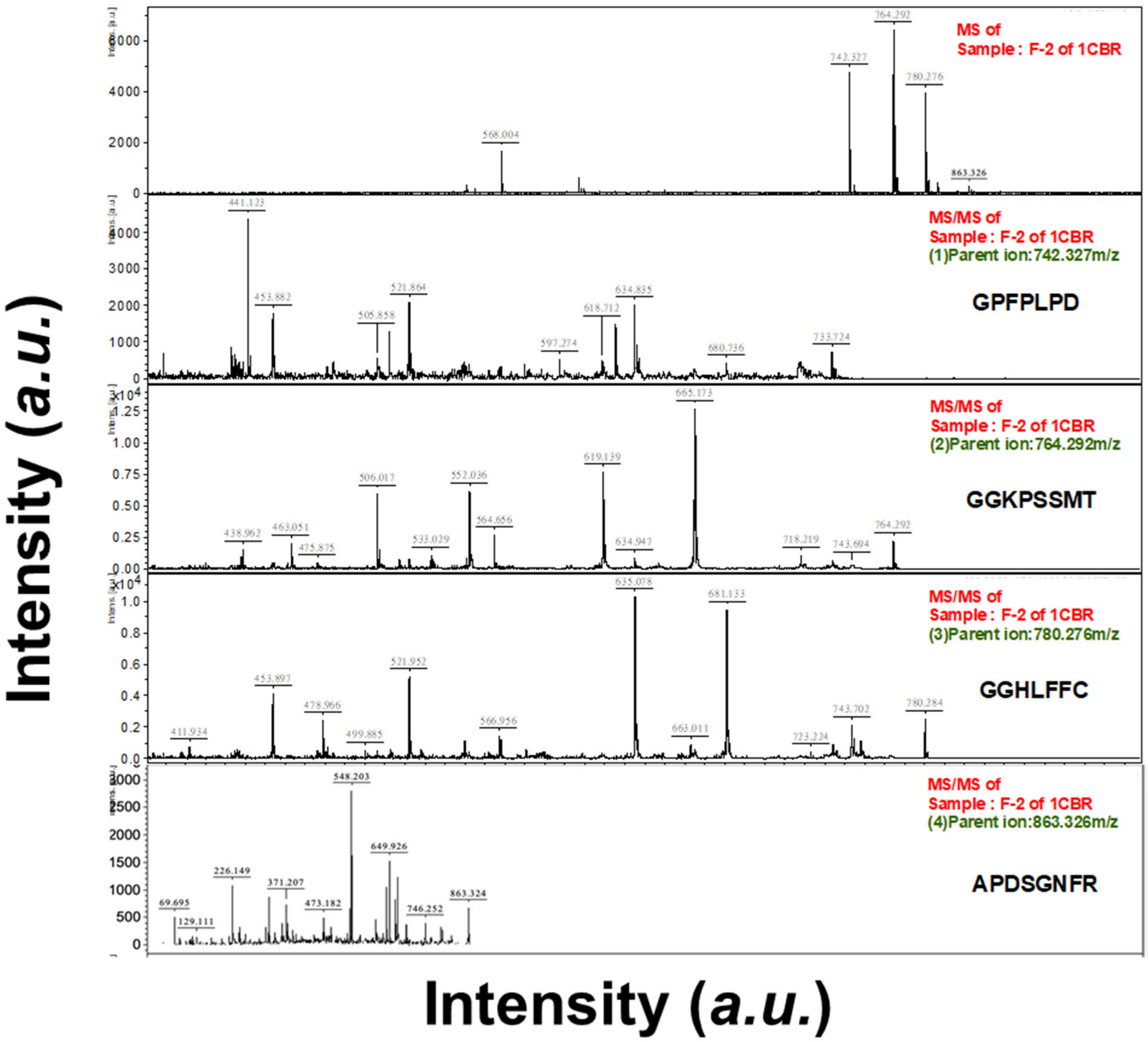

Figure. S2. MALDI-TOF MS analysis of Fraction 2 (F2) revealed four predominant peptide sequences: GPFPLPD, GGKPSSMT, GGHLFFC, and APDSGNFR. The mass spectrum illustrates the major ion peaks corresponding to these peptides.

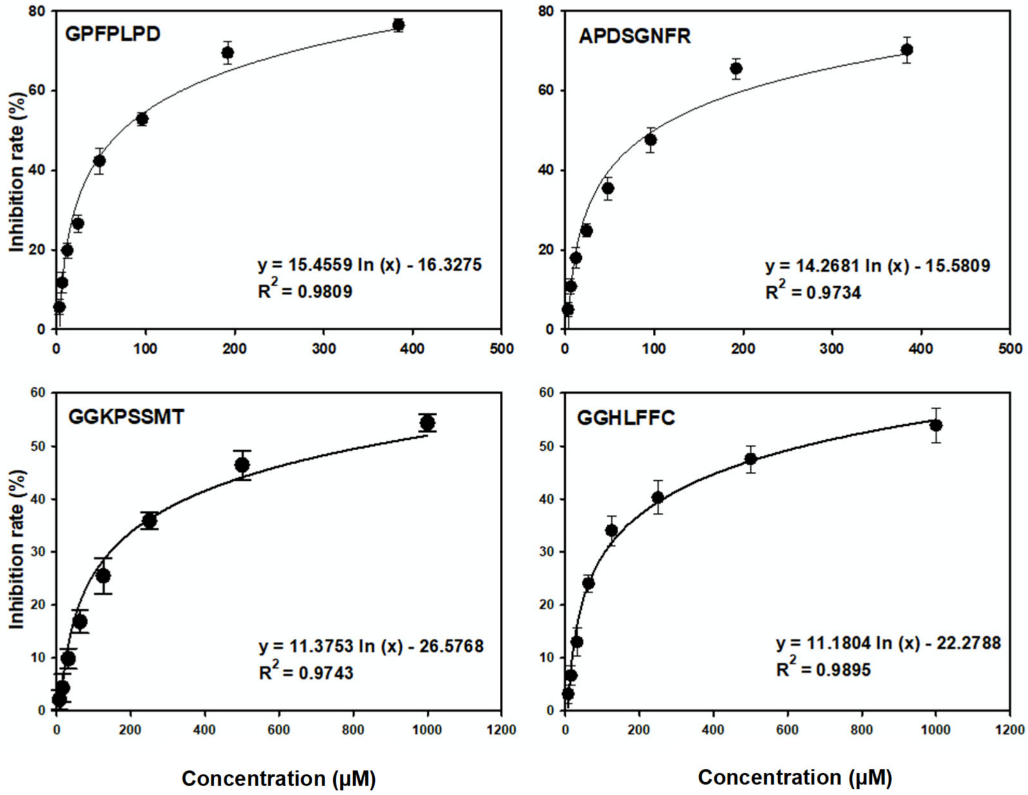

Figure. S3. Enzymatic inhibition kinetics of DPP-IV in the presence of synthesized peptides. The kinetic behavior was evaluated using H-Gly-Pro-AMC as a fluorogenic substrate in 100 mM Tris-HCl buffer (pH 7.0) at 37°C. Peptides were tested at various concentrations (0–1000 μM), and initial reaction velocities were determined based on fluorescence intensity (Ex/Em: 382/460 nm). Lineweaver–Burk plots were used to analyze the inhibition pattern. All experiments were performed in triplicate, and data are presented as mean ± SD.

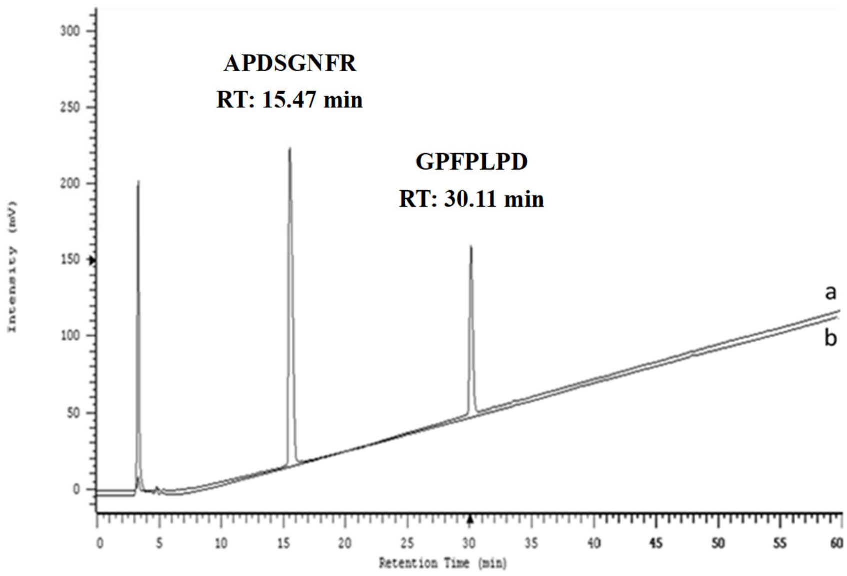

Figure. S4. The elution profiles of the synthetic peptides GPFPLPD (designated as peak a) and APDSGNFR (designated as peak b) were characterized by reversed-phase high-performance liquid chromatography (RP-HPLC). Chromatographic separation was conducted on a C18 column using a linear gradient elution of solvent B (0.1% trifluoroacetic acid in acetonitrile) from 5% to 40% over 60 minutes at a constant flow rate of 0.7 mL/min, with solvent A comprising distilled deionized water. Detection of peptides was performed by monitoring absorbance at 220 nm.

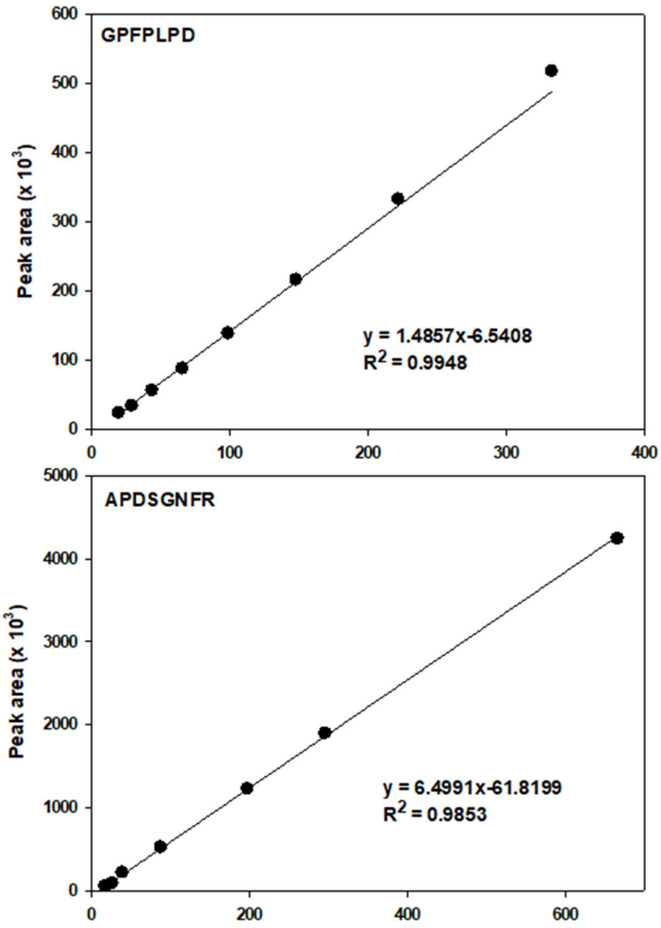

Figure. S5. Calibration curves of the synthetic peptides GPFPLPD and APDSGNFR for quantitative analysis in the rat absorption study. The peptides were analyzed by RP-HPLC using a linear gradient of solvent B (0.1% TFA in acetonitrile) from 5% to 40% over 60 minutes at a flow rate of 0.7 mL/min. Solvent A consisted of distilled deionized water. Elution was monitored at 220 nm. The resulting calibration curves were used to quantify peptide concentrations in plasma samples collected from Sprague-Dawley rats.
